# Bone Marrow Mesenchymal Stem Cell‐Derived Exosomal Let‐7b‐5p Reduces High Glucose‐Induced Microglial Activation and Inflammation Through TLR4/ATF4

**DOI:** 10.1155/mi/7251718

**Published:** 2026-02-10

**Authors:** Yepin Zhang, Yiyi Luo, Jian Han, Ling Wang, Hong Xu, Libo Zhang, Peiqi Chen, Heng Luo

**Affiliations:** ^1^ Department of Pathology, The People’s Hospital of Chuxiong Yi Autonomous Prefecture and The Fourth Affiliated Hospital of Dali University, Chuxiong, China; ^2^ Precision Medicine Center of Chuxiong Yi Autonomous Prefecture, The People’s Hospital of Chuxiong Yi Autonomous Prefecture and The Fourth Affiliated Hospital of Dali University, Chuxiong, China; ^3^ Department of Endocrinology, The People’s Hospital of Chuxiong Yi Autonomous Prefecture and The Fourth Affiliated Hospital of Dali University, Chuxiong, China; ^4^ Department of Ophthalmology, The People’s Hospital of Chuxiong Yi Autonomous Prefecture and The Fourth Affiliated Hospital of Dali University, Chuxiong, China

**Keywords:** bone marrow mesenchymal stem cells, diabetic retinopathy, exosomes, let-7b-5p, microglial cell activation, TLR4/ATF4 pathway

## Abstract

**Background and Objective:**

Diabetic retinopathy (DR) is a leading cause of vision loss in patients with diabetes mellitus (DM), and its pathogenesis is closely associated with aberrant microglial activation. Although bone marrow mesenchymal stem cell–derived exosomes (BMSC‐Exo) and the miRNAs that they carry show therapeutic potential for DR, the specific roles and molecular mechanisms through which let‐7b‐5p regulates microglial activation after it is delivered by BMSC‐Exo remain unclear. This study aimed to elucidate the function and underlying mechanism of BMSC‐Exo let‐7b‐5p in DR.

**Methods:**

A DR mouse model was established by intraperitoneal injection of streptozotocin (STZ), and BV‐2 microglia were stimulated with high glucose (HG) to induce activation in vitro. The morphological characteristics of the BMSC‐Exo were identified using transmission electron microscopy (TEM). Protein and gene expression levels, as well as microglial activation, were assessed by Western blot, RT‐qPCR, and immunofluorescence, respectively. Retinal tissue damage and apoptosis were evaluated using HE staining and TUNEL assays.

**Results:**

BMSC‐Exo treatment significantly suppressed the expression of activation markers (Iba1 and TSPO) and inflammatory cytokines (TNF‐α, IL‐1β, and IL‐6) in HG‐induced BV‐2 cells and DR mouse retinas while alleviating retinal tissue damage and apoptosis. Bioinformatics analysis revealed the downregulation of let‐7b‐5p in DR. Functional experiments demonstrated that let‐7b‐5p overexpression enhanced the inhibitory effects of BMSC‐Exo on microglial activation, inflammation, and retinal damage, whereas let‐7b‐5p knockdown attenuated these therapeutic benefits. Mechanistically, BMSC‐Exo let‐7b‐5p inhibited excessive microglial activation and inflammatory responses by targeting the TLR4/ATF4 signaling pathway.

**Conclusion:**

BMSC‐Exo deliver let‐7b‐5p to suppress the TLR4/ATF4 pathway, thereby mitigating microglial activation and inflammation and ultimately delaying DR progression. These findings support the potential of this novel therapeutic strategy for targeted DR treatment.

## 1. Introduction

Diabetes mellitus (DM) poses a significant public health issue that impacts global health. Recent alterations in dietary patterns and lifestyle habits have led to an increase in the occurrence of DM [1]. Globally, diabetic retinopathy (DR) is the primary eye‐related complication linked to DM and is the foremost cause of blindness among the workforce [2]. DR can result in issues such as retinal ischemia, bleeding, the formation of new blood vessels in the retina, and swelling of the macula [3]. Furthermore, reports indicate that microglia significantly contribute to the progression of DR [4, 5]. Microglia play a crucial role as inherent immune cells in the retina and are typically situated in the inner layer, aiding in the surveillance and elimination of retinal metabolites [6]. During the progression of DR, localized retinal inflammation triggers microglia in the retinal plexiform layer to transition from a monitoring state to an active state. Such a phenotypic alteration aids in counteracting harmful stimuli and reestablishing tissue balance [7]. Nonetheless, the ongoing stimulation of microglia leads to the generation of various inflammatory agents (such as cytokines and chemokines), thereby accelerating the progression of DR [8]. Consequently, focusing on the suppression of microglial activation and inflammation serves as a potent strategy for treating DR.

Bone marrow mesenchymal stem cells (BMSCs) are a type of stem cell capable of various differentiation processes. Within the field of regenerative medicine, BMSCs are among the most prevalently utilized cells in laboratory studies and clinical experiments [9]. Earlier research has indicated that administering BMSCs intravitreally serves as an efficient method for promoting the repair of damaged intraretinal blood vessels and nerve cells [10]. Recent research has verified that stem cell‐secreted bioactive elements and microvesicles (EVs) play key roles in the therapeutic effects of stem cells [11, 12]. Within this group, exosomes (Exo) stand out as the most diminutive form of EVs, encompassing DNA and RNA strands, lipid elements, metabolites, proteins, and various other components [13]. The secretion of EVs is crucial for many biological functions, including communication between cells and the maintenance of homeostasis [14]. Recent research has indicated that bone marrow mesenchymal stem cell–derived exosomes (BMSC‐Exo) can serve as a potent treatment for DR [15, 16].

Notably, miRNAs conveyed through BMSC‐Exo are crucial agents in the progression of DR [17]. miRNAs, a category of noncoding RNA, span ~22 nucleotides in length. These elements control gene expression by binding to the untranslated areas of target mRNAs, significantly influencing the regulation of human physiological and pathological functions [18]. In a study by El‐Halim [19], miR‐16 and miR‐100 contained in mesenchymal stem cell (MSC)–derived Exo inhibited angiogenesis in DR by targeting VEGF. Similarly, Exo derived from adipose‐derived MSCs enhanced DR by suppressing inflammation and angiogenesis via the release of miR‐192 [20]. Recent research has indicated that MSC‐Exo can also release let‐7b‐5p [21]. The role of let‐7b‐5p in regulating angiogenesis [22] and inflammatory responses [23] has already been reported. Furthermore, a link between the reduced expression of let‐7b‐5p and the emergence of DM has been verified [24]. Interestingly, Liu et al. [25] reported that let‐7b‐5p, which originates from Exo in induced pluripotent stem cell‐derived neural stem cells (iPSC‐NSCs), can alleviate spinal cord injury by suppressing microglial pyroptosis and neuroinflammation. However, the process through which BMSC‐Exo influence DR via the ability of let‐7b‐5p to control microglial activation and inflammation remains unexplored.

Toll‐like receptors (TLRs) function as pattern recognition receptors and significantly influence innate immunity [26]. TLR4, which belongs to the TLR family, functions as a transmembrane protein, identifying extracellular antigens or pathogens and transporting them into cells [27]. TLR4 activation subsequently triggers the activation of downstream genes and the secretion of substantial quantities of inflammatory cytokines (such as IL‐6 and IL‐1β), leading to inflammation [28]. TLR4‐mediated microglial activation and inflammation are also key factors contributing to the progression of DR [29]. Furthermore, activating transcription factor 4 (ATF4), a member of the ATF/CREB family, plays a role in various biological functions, including stress reactions, bone absorption, blood formation in the medulla, and lens development [30]. Notably, it has been demonstrated that ATF4 plays a role in the TLR4‐driven innate immune reaction among human monocytes [31]. In addition, Gong et al. [32] reported that let‐7b‐5p released by serum Exo is capable of suppressing macrophage activation and inflammation through the suppression of the TLR4 signaling pathway. These findings indicate the potential existence of a let‐7b‐5p/TLR4/ATF4 signaling pathway in DR that controls the activation of microglia and inflammation.

To summarize, the objective of this research was to explore how let‐7b‐5p transported in BMSC‐Exo influences microglial activation and inflammation and its impact on DR progression.

## 2. Materials and Methods

### 2.1. Construction of and Intervention in the DR Mouse Model

Twenty‐four male C57BL/6 mice, aged 8 weeks and weighing between 20 and 24 g, were acquired from Hunan SJA Laboratory Animal Co., Ltd. Mice were randomly assigned to groups by a researcher not involved in experiments or data analysis. During the injection, treatment assessment, and data analysis phases, all operators and assessors were blinded to group allocation by using coded samples. Following 1 week of acclimatization, a total of 18 mice were utilized to create the DR model. The DR model construction method was as described by Wang et al. [33]. In detail, the mice received daily intraperitoneal injections of 55 mg/kg streptozotocin (STZ) over a span of 5 days, in contrast to the 6 mice from the control group, which were administered an identical volume of PBS intraperitoneally. Blood glucose levels were monitored starting 7 days after the first STZ injection, and mice with blood glucose levels > 16.7 mmol/L were considered to have DM. Eighteen mice with diabetes were subsequently divided into three equal groups, and the indicated treatments were administered following the methods of Liu et al. [34]. In the DR + BMSC‐Exo group, mice received an injection of 1 μL of BMSC‐Exo into their vitreous cavity; for the DR + agomir‐let‐7b‐5p‐Exo group, mice were given 1 μL of Exo derived from BMSCs overexpressing let‐7b‐5p (agomir‐let‐7b‐5p), and for the DR + antagomir‐let‐7b‐5p‐Exo group, 1 μL of Exo derived from BMSCs with knockdown of let‐7b‐5p (antagomir‐let‐7b‐5p) was injected. Exo, at a concentration of 5 μg/μL, were administered every 3 days over a 6‐week intervention duration, whereas mice in the DR group received an identical amount of PBS in their vitreous cavity. For intravitreal injections, mice were anesthetized by inhalation of 3%–4% isoflurane and maintained on 1.5%–2% isoflurane. After the 6‐week intervention, all mice were euthanized via an intraperitoneal injection of pentobarbital sodium (150 mg/kg), and serum and retinal tissues were collected for subsequent experiments. All the above experiments were approved by the Ethics Committee of Chuxiong Yi Autonomous Prefecture People’s Hospital (No. 2022‐11) and were performed in accordance with the ARRIVE guidelines for the care and use of laboratory animals.

### 2.2. Cell Culture and Transfection

In this study, mouse BMSCs (mBMSCs, CP‐M131, tissue origin: bone marrow) and the mouse microglial cell line BV‐2 (CL‐0493, RRID: CVCL_0182; tissue origin: brain, glioma; species: *Mus musculus*; strain: C57BL/6; sex: female) were purchased from Procell Life Science & Technology Co., Ltd. (Wuhan, China) in 2024. The BV‐2 cell line was authenticated by short tandem repeat (STR) profiling and is not known to be misidentified or contaminated. mBMSCs were isolated from mouse bone marrow tissue, with a purity exceeding 90%, as verified by CD29 or CD44 immunofluorescence staining, and confirmed to be mycoplasma free through contamination testing. In accordance with the guidelines provided by the manufacturer, mBMSCs were grown in complete mBMSCs medium (CM‐M131; Pricella, China), with the medium replaced every 2–3 days. BV‐2 cells were cultivated in DMEM (11965092; Gibco, USA) supplemented with 10% fetal bovine serum (A5256701; Gibco, USA) and 1% penicillin‒streptomycin (C0222; Beyotime, China). In accordance with the experimental requirements, the BV‐2 cells were divided into three groups for culture: cells cultured in normal glucose (NG, 5.5 mM glucose) medium served as the baseline control, cells cultured in medium containing normal glucose along with 25 mM mannitol served as the osmotic control, and cells cultured in high glucose (HG, 25 mM glucose) medium were used to establish a HG‐induced inflammatory model. Every cell culture used in this research was incubated at a temperature of 37 °C in an environment containing 5% CO_2_.

To elucidate the specific role of the TLR4/ATF4 signaling axis in microglial activation, the TLR4‐specific activator CRX‐527 (HY‐155801; MedChemExpress, USA) was introduced into BV‐2 cells, and the ATF4 gene was knocked down (si‐ATF4). With the use of Lipofectamine 3000 transfection reagent (L3000150; Invitrogen, USA), BV‐2 cells were transfected with si‐ATF4. After transfection, the cells were cultured at 37 °C for an additional 48 h, and the expression level of the ATF4 protein was subsequently detected by Western blotting to evaluate the knockdown efficiency.

To investigate the effects of let‐7b‐5p on BV‐2 activation in mBMSCs, Lipofectamine 3000 transfection reagent (L3000150; Invitrogen, USA) was used to transfect mBMSCs with let‐7b‐5p overexpression (agomir‐let‐7b‐5p) and overexpression control (agomir‐NC) sequences, as well as let‐7b‐5p knockdown (antagomir‐let‐7b‐5p) and knockdown control (antagomir‐NC) sequences. The transfection efficiency was then verified by detecting let‐7b‐5p expression in the cell line using RT‐qPCR.

### 2.3. mBMSCs Differentiation Experiment

To assess the potential of mBMSCs to differentiate into osteogenic and adipogenic forms, these cells were grown in either mBMSCs osteogenic differentiation medium (PD‐003; Pricella, China) or adipogenic differentiation medium (PD‐004; Pricella, China). The medium was replaced with fresh differentiation medium every 3 days. Following a 3‐week period of differentiation induction, the medium was discarded, the cells were washed three times with 1 × PBS, and the cells were then fixed using 4% neutral formalin for 15 min. Osteoblasts and adipoblasts were stained with Alizarin red S (ARS) and Oil red O for half an hour, rinsed three times with PBS, and examined under a microscope (Nikon, Japan).

### 2.4. Identification of mBMSCs Surface Markers via Flow Cytometry

To identify surface markers on mBMSCs by flow cytometry, the cells were resuspended in PBS to achieve a density of 1 × 10^6^ cells. The cells were subsequently incubated with FITC‐conjugated primary antibodies (Invitrogen, USA) against CD29 (1:200, 11‐0291‐82), CD44 (1:200, 11‐0441‐81), CD90 (1:200, 11‐0909‐42), and CD45 (1:200, 11‐0451‐82) in the dark at 37 °C for 30 min. After incubation, the expression of CD29, CD44, CD90, and CD45 in mBMSCs was analyzed using flow cytometry.

### 2.5. Isolation and Characterization of mBMSC‐Derived Exo (mBMSCs‐Exo)

When the cell density of the mBMSCs reached 80%, the cells were resuspended in exosome‐free medium and cultured for 48 h, after which the culture medium was collected. The supernatant was subsequently filtered through a 0.22‐μm filter and concentrated using an ultrafiltration tube. Following the instructions of the ExoQuick‐TC exosome precipitation kit (EXOTC10A‐1; System Biosciences, USA), the reagent and concentrated supernatant were mixed at the appropriate ratio and incubated. Finally, the mixture was centrifuged at 10,000 × *g* to pellet the Exo. Next, the morphology of the Exo was observed by transmission electron microscopy (TEM). Moreover, Western blotting was used to detect the expression of the exosomal markers CD63, Alix, and TSG101.

### 2.6. Western Blot Analysis

Total protein was extracted from retinal tissues, BV‐2 cells, and Exo using advanced RIPA lysis buffer (R0010, Solarbio, China). The protein concentration was measured using a BCA protein concentration assay kit (P0010; Beyotime, China). Afterward, equal amounts of protein were separated by SDS‒PAGE and transferred onto a polyvinylidene difluoride (PVDF) membrane (88518; Thermo Fisher, USA). The membrane was incubated overnight at 4 °C with the following diluted primary antibodies: anti‐CD63 (1:1000; ab315108; Abcam, UK), anti‐Alix (1:1000; ab275377; Abcam, UK), anti‐TSG101 (1:1000; ab125011; Abcam, UK), anti‐Iba1 (1:1000; ab178846; Abcam, UK), anti‐TSPO (1:2000; ab109497; Abcam, UK), anti‐TLR4 (1:1000; 19811‐1‐AP; Proteintech, China), anti‐ATF4 (1:1000; ab216839; Abcam, UK), and anti‐β‐actin (1:1000; ab8226; Abcam, UK). The membrane was then incubated with the corresponding secondary antibody at room temperature for 1 h. Color development was performed using an enhanced chemiluminescence (ECL) kit (WP20005; Invitrogen, USA), and the bands were semiquantitatively analyzed using ImageJ software (NIH, Bethesda, USA).

### 2.7. Uptake of Exo by BV‐2 Cells

Exo were fluorescently labeled by coincubation with a 4 mg/mL PKH67 solution (PKH67GL‐1KT; Sigma‒Aldrich, USA). Excess dye was subsequently removed by centrifugation at 100,000 g and 4 °C, after which the labeled Exo were washed three times with PBS. The PKH67‐labeled Exo were then cultured with BV‐2 cells at 37 °C for 24 h. The cells were then washed with PBS, fixed with 4% paraformaldehyde for 15 min, and observed under a confocal laser scanning microscope to examine the cellular uptake of the Exo.

### 2.8. Immunofluorescence

Cells or tissue sections were fixed with 4% paraformaldehyde, permeabilized with 0.3% Triton X‐100, and blocked with 5% bovine serum albumin (BSA). The samples were subsequently incubated overnight at 4 °C with primary antibodies against Iba1 (1:100; ab178846; Abcam, UK) and TSPO (1:100; ab109497; Abcam, UK). After incubation, the samples were treated with the corresponding secondary antibodies and DAPI, followed by imaging under a fluorescence microscope (Nikon, Japan).

### 2.9. ELISA

The concentrations of TNF‐α, IL‐1β, and IL‐6 in the supernatants of BV‐2 cells and in mouse serum were measured with the corresponding kits (TNF‐α: SEKM‐0034; IL‐1β: SEKM‐0002; IL‐6: SEKM‐0007) from Solarbio (China) following the manufacturer’s instructions. The absorbance was read at 450 nm, and cytokine levels were determined on the basis of standard curves.

### 2.10. Real‐Time Fluorescence Quantitative PCR

Following total RNA extraction (PureLink miRNA Isolation Kit; K157001; Invitrogen, USA) from mBMSCs, BV‐2 cells, and retinal tissue, cDNA was synthesized (BeyoRT III Kit; D7178; Beyotime, China). RT‐qPCR analysis was conducted with BeyoFast SYBR Green qPCR Mix (D7260; Beyotime, China), and U6 was used as an endogenous control for normalization. The relative gene expression was determined by the 2^*−ΔΔCt*^ method. All primer sequences are listed in Table 1.

**Table 1:** Primer sequences.

Gene	Sequence (F: forward primer; R: reverse primer)
let‐7b‐5p	F: 5´‐GCGCGTGAGGTAGTAGGTTGT‐3´
	R: 5´‐AGTGCAGGGTCCGAGGTATT‐3´
U6	F: 5´‐CTCGCTTCGGCAGCACA‐3´
	R: 5´‐AACGCTTCACGAATTTGCGT‐3´

### 2.11. Dual‐Luciferase Reporter Gene Experiments

On the basis of the predicted binding site between let‐7b‐5p and TLR4, a fragment of the TLR4 3′‐UTR containing the let‐7b‐5p binding site was cloned and inserted into the pGL3 vector (Promega) to construct the wild‐type TLR4 reporter plasmid (TLR4 WT). A mutant TLR4 reporter plasmid (TLR4 MUT) was subsequently generated using a Phusion Site‐Directed Mutagenesis Kit (F541; Thermo Fisher Scientific, USA). HEK‐293T cells were then cotransfected with either the TLR4 WT or MUT reporter plasmid together with agomir‐let‐7b‐5p or a negative control using Lipofectamine 3000 (L3000150; Invitrogen, USA). Forty‐eight hours after transfection, luciferase activity was measured using a dual‐luciferase reporter assay system (Promega).

### 2.12. HE Staining

After routine dewaxing, the tissue sections were stained with hematoxylin for 5 min at room temperature. The sections were then rinsed twice with running tap water (2 min per wash), followed by counterstaining with eosin solution for 1–2 min. After another two washes with tap water (2 min each), the sections were dehydrated through a graded ethanol series, cleared in xylene I (5 min) and xylene II (5 min), mounted with neutral balsam, and imaged under a light microscope (Nikon, Japan).

### 2.13. TUNEL Staining

Retinal tissue sections were washed twice with PBS, after which 50 µL of TUNEL detection solution (containing 5 µL of TdT enzyme and 45 µL of fluorescein‐labeled dUTP; C1089; Beyotime, China) was added to each section. The sections were then incubated at 37 °C in the dark for 1 h. After incubation, the sections were mounted with antifade mounting medium and imaged under a fluorescence microscope (Nikon, Japan).

### 2.14. Statistical Analysis

All experimental data are presented as the mean ± standard deviation (SD). Statistical analyses were performed using GraphPad Prism 8.0.1. Data comparisons between two groups were conducted using Student’s *t*‐test, while comparisons among multiple groups were carried out using one‐way analysis of variance (ANOVA), followed by Tukey’s post hoc test for multiple comparisons. A *p*‐value of < 0.05 was considered to indicate statistical significance.

## 3. Results

### 3.1. Characterization of the mBMSCs and mBMSCs‐Exo

First, we identified mBMSCs and mBMSCs‐Exo. Microscopic examination revealed that mBMSCs exhibited a tightly arranged fibroblast‐like morphology with elongated spindle shapes after adherence (Figure 1A). Second, we assessed the differentiation potential of mBMSCs through specific induction assays and observed the formation of calcium deposits (Figure 1B) and lipid droplets (Figure 1C) in mBMSCs cultures. We then analyzed the expression of surface markers on mBMSCs by flow cytometry and detected high expression of CD29, CD44, and CD90 but low expression of CD45 (Figure 1D). This expression profile is characteristic of BMSCs [35]. The above results confirm that the cells we cultured were BMSCs. We subsequently examined the features of the Exo by TEM and revealed that the mBMSCs‐Exo appeared as round vesicles (Figure 1E). Additionally, the exosomal marker proteins CD63, Alix, and TSG101 were highly expressed in mBMSCs‐Exo (Figure 1F). These findings indicate that Exo were successfully isolated from mBMSCs.

Figure 1Characterization of the mBMSCs and mBMSCs‐Exo. (A) Morphology of mBMSCs. (B) Alizarin red staining after osteogenic differentiation. (C) Oil red O staining after adipogenic differentiation. (D) Flow cytometric analysis of the expression of the mBMSCs surface markers CD29, CD44, CD90, and CD45. (E) Morphology of the exosomes observed by TEM, scale bar: 100 nm. (F) Western blot detection of the exosomal markers CD63, Alix, and TSG101.

**Figure:**
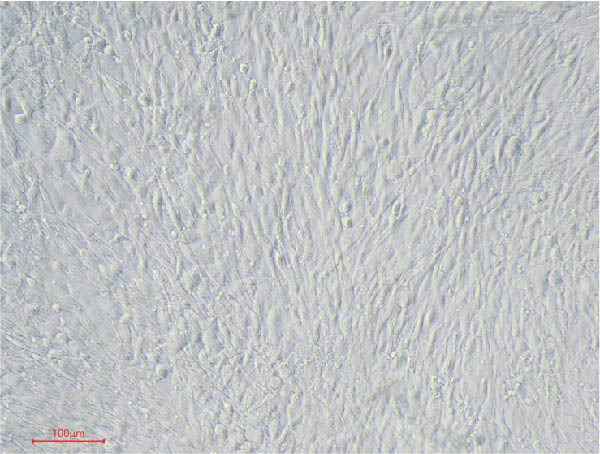
(A)

**Figure:**
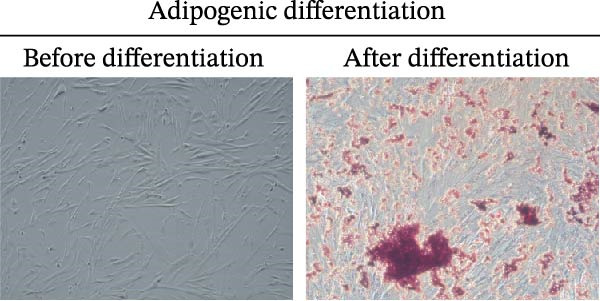
(B)

**Figure:**
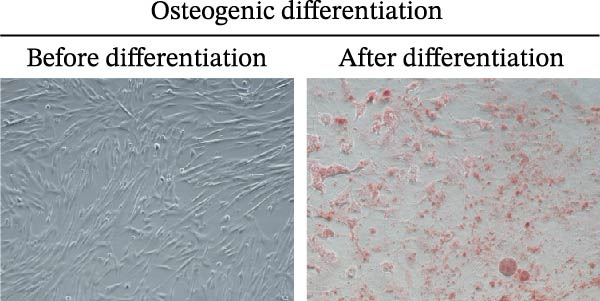
(C)

**Figure:**
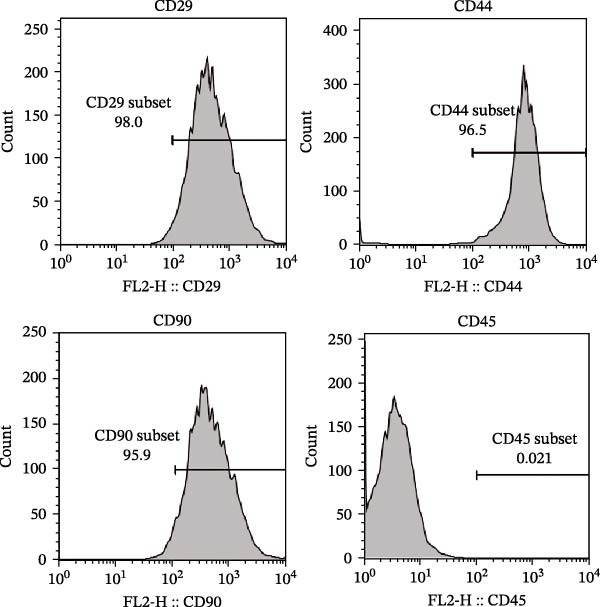
(D)

**Figure:**
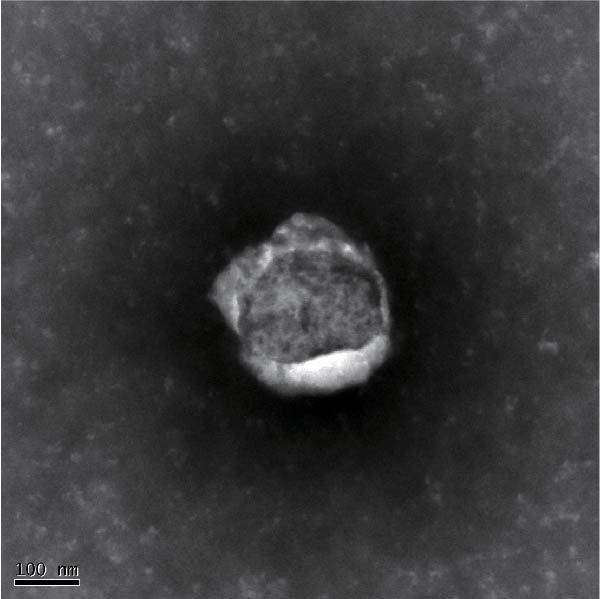
(E)

**Figure:**
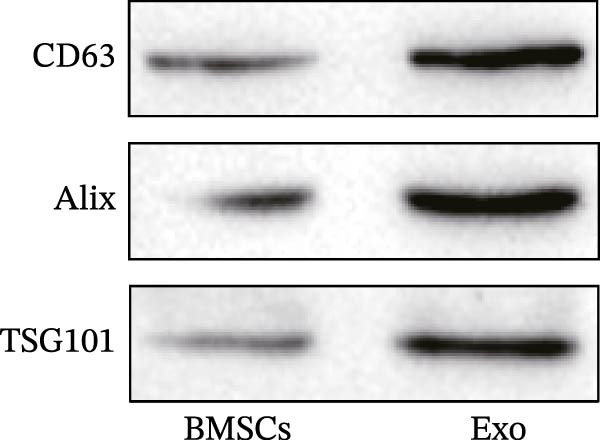
(F)

### 3.2. mBMSCs‐Exo Inhibited HG‐Induced Microglial Activation and Inflammation

Previous studies have indicated that BMSCs‐Exo are capable of controlling microglial activation and inflammation during cerebral ischemia‒reperfusion injury [36]. Therefore, we explored the effects of mBMSCs‐Exo on HG‐induced microglial activation and inflammation. First, BV‐2 cells were incubated with PKH67‐labeled mBMSCs‐Exo. Fluorescence microscopy revealed fluorescence signals within the BV‐2 cells, indicating successful uptake of mBMSCs‐Exo by the BV‐2 cells (Figure 2A). Next, we evaluated the effect of mBMSCs‐Exo on the HG‐induced activation of BV‐2 cells. Western blot analysis revealed that HG treatment upregulated the expression of Iba1 and TSPO in BV‐2 cells, but these effects were suppressed by additional treatment with mBMSCs‐Exo. No significant differences were detected between the NG group and the mannitol osmotic control group (Figure 2B). Similarly, immunofluorescence experiments revealed that mBMSCs‐Exo partially attenuated the HG‐induced upregulation of Iba1 and TSPO expression in BV‐2 cells, with the mannitol group showing expression levels comparable to those in the NG group (Figure 2C,D). Furthermore, treatment with mBMSCs‐Exo effectively reversed the HG‐induced increase in the levels of the inflammatory cytokines TNF‐α, IL‐1β, and IL‐6, whereas the cytokine levels in the mannitol group were similar to those in the NG group (Figure 2E–G). These results indicate that mBMSCs‐Exo can inhibit HG‐induced microglial activation and inflammatory responses independent of osmotic effects.

Figure 2mBMSCs‐Exo inhibited HG‐induced microglial activation and inflammation. (A) Uptake of PKH67‐labeled mBMSCs‐Exo by BV‐2 cells, scale bar: 10 μm. (B) Expression levels of Iba1 and TSPO in BV‐2 cells detected by Western blot. (C,D) Expression levels of Iba1 and TSPO in BV‐2 cells detected by immunofluorescence, scale bar: 10 μm. (E–G) Levels of the inflammatory cytokines TNF‐α, IL‐1β, and IL‐6 in the supernatant of BV‐2 cells measured by ELISA. Compared with the NG group,  ^∗∗∗^ *p* < 0.001; compared with the HG group, ^###^ *p*  < 0.001.

**Figure:**
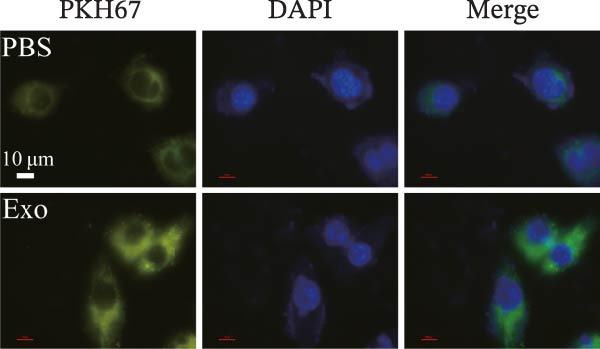
(A)

**Figure:**
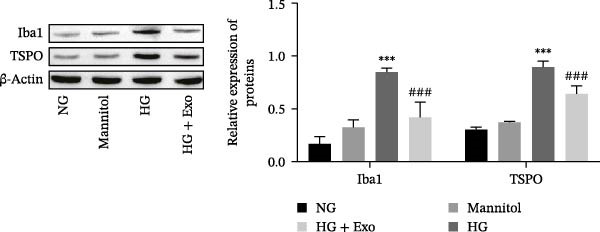
(B)

**Figure:**
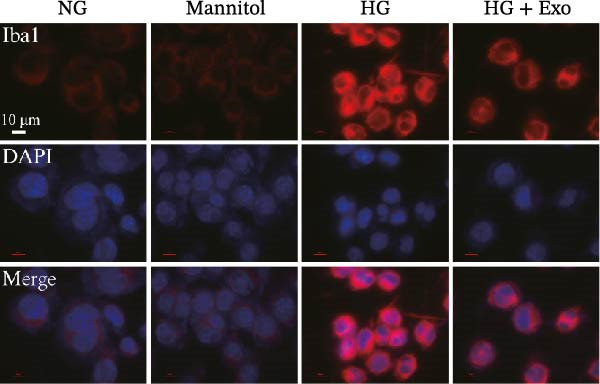
(C)

**Figure:**
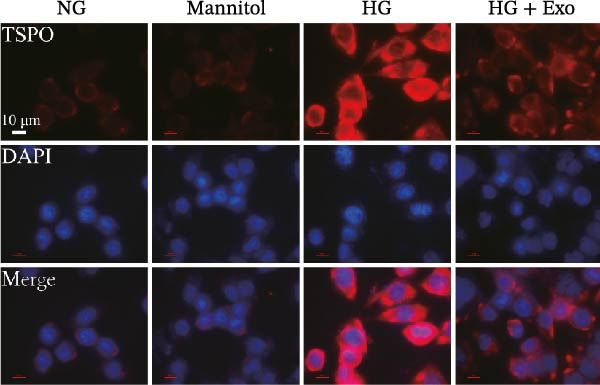
(D)

**Figure:**
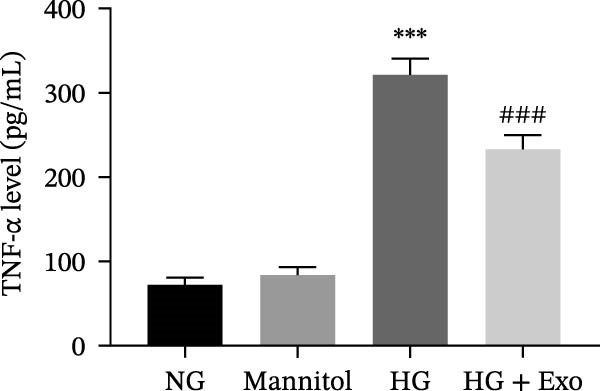
(E)

**Figure:**
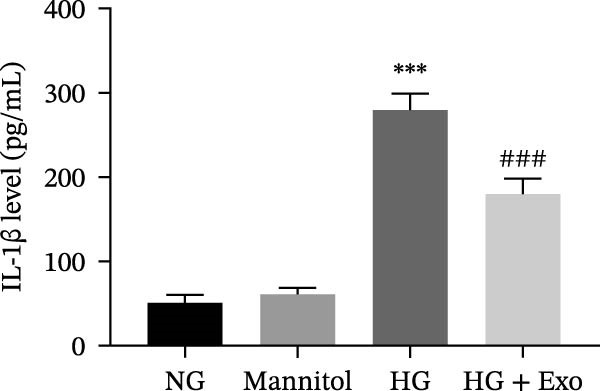
(F)

**Figure:**
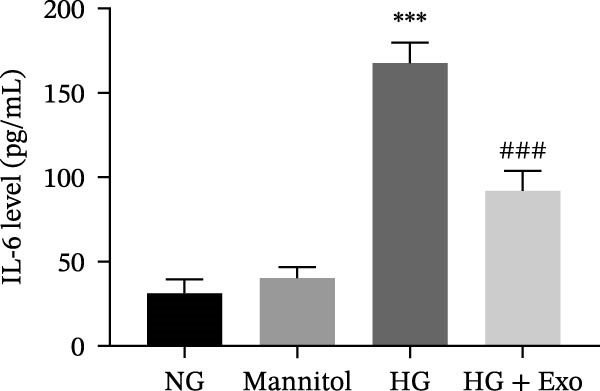
(G)

### 3.3. mBMSCs‐Exo Suppress HG‐Induced Microglial Activation and Inflammation Through the Inhibition of TLR4/ATF4

Previous studies have indicated that activation of the TLR4/ATF4 signaling axis can induce an inflammatory phenotype in human monocytes [31]. Therefore, the current study aimed to investigate whether the inhibitory effect of mBMSCs‐Exo on HG‐induced microglial activation and inflammation is mediated through the regulation of the TLR4/ATF4 signaling axis. Western blot results revealed that HG treatment significantly upregulated the expression of TLR4 and ATF4 in BV‐2 cells, whereas mBMSCs‐Exo intervention decreased their expression levels (Figure 3A). To further validate the role of this signaling axis, we performed experiments using the TLR4 agonist CRX‐527 and ATF4 knockdown (si‐ATF4). First, si‐ATF4 effectively reduced ATF4 expression, confirming its successful knockdown (Figure S1A). Subsequent protein analysis revealed that HG markedly increased the expression of both TLR4 and ATF4, whereas mBMSCs‐Exo significantly suppressed their expression. Treatment with the TLR4 agonist CRX‐527 partially reversed these inhibitory effects, restoring the expression levels of TLR4 and ATF4. In contrast, ATF4 knockdown suppressed ATF4 expression but did not significantly affect TLR4 expression (Figure 3B). Immunofluorescence staining and ELISA further demonstrated that CRX‐527 attenuated the therapeutic effect of mBMSCs‐Exo by partially restoring the expression of Iba1 and TSPO in BV‐2 cells and increasing the secretion levels of TNF‐α, IL‐1β, and IL‐6. Conversely, ATF4 knockdown diminished the effect of CRX‐527 (Figure 3C–G). In all the experiments, the mannitol osmotic control group did not significantly differ from the NG group, indicating that mBMSCs‐Exo inhibit HG‐induced microglial activation and inflammation via the TLR4/ATF4 signaling axis, independent of any osmotic effects.

Figure 3mBMSCs‐Exo suppress HG‐induced microglial activation and inflammation through the inhibition of TLR4/ATF4. (A) TLR4 and ATF4 levels in BV‐2 cells were determined through Western blotting. (B) Western blotting was used to measure the expression of TLR4 and ATF4 in BV‐2 cells. (C, D) Immunofluorescence staining for the expression of Iba1 and TSPO in BV‐2 cells, scale bar: 10 μm. (E–G) ELISA was used to measure the levels of the inflammatory cytokines TNF‐α, IL‐1β, and IL‐6 in the supernatant of BV‐2 cells. Compared with the NG group,  ^∗∗∗^ *p*  < 0.001; compared with the HG group, ^##^ *p*  < 0.01 and ^###^ *p*  < 0.001; compared with the HG+ Exo group, ^&&&^ *p*  < 0.001; compared with the HG+ Exo+ CRX‐527 group, ^^^ *p*  < 0.05 and ^^^^^ *p*  < 0.001.

**Figure:**
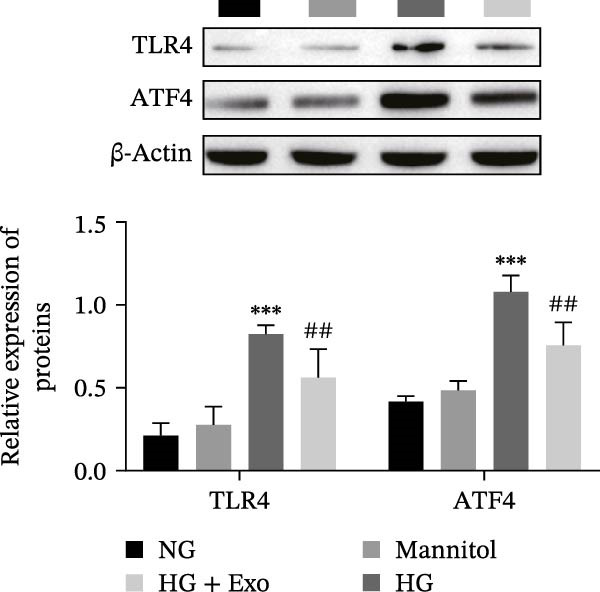
(A)

**Figure:**
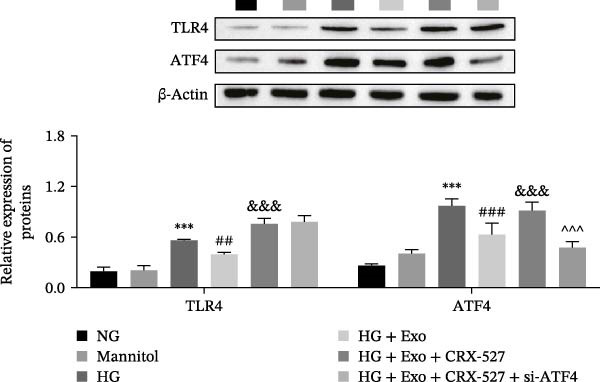
(B)

**Figure:**
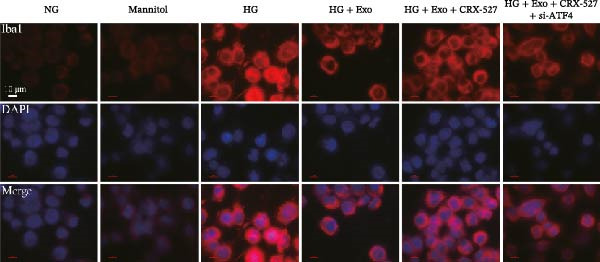
(C)

**Figure:**
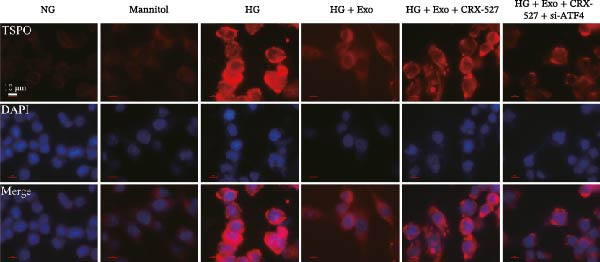
(D)

**Figure:**
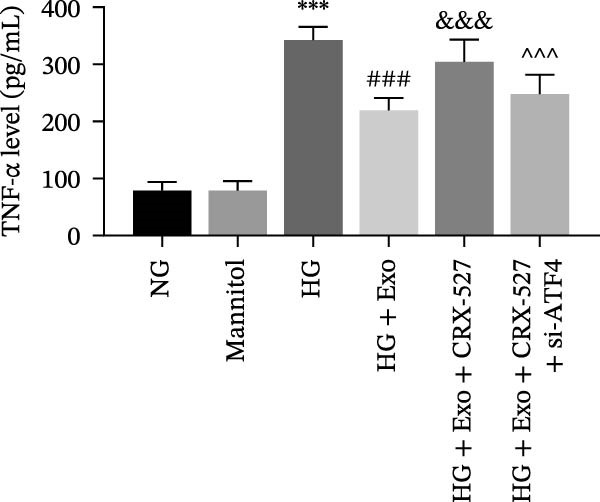
(E)

**Figure:**
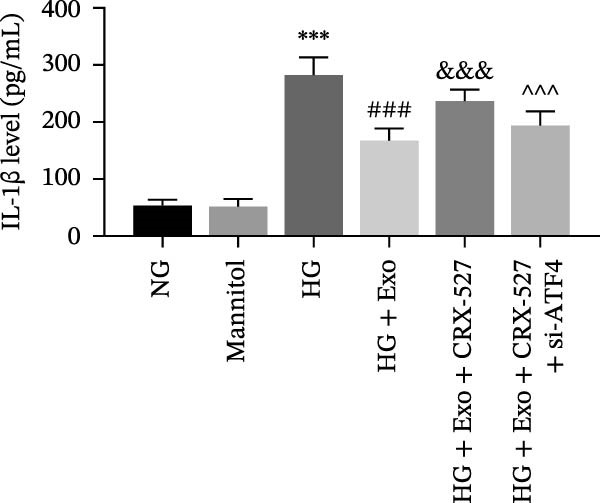
(F)

**Figure:**
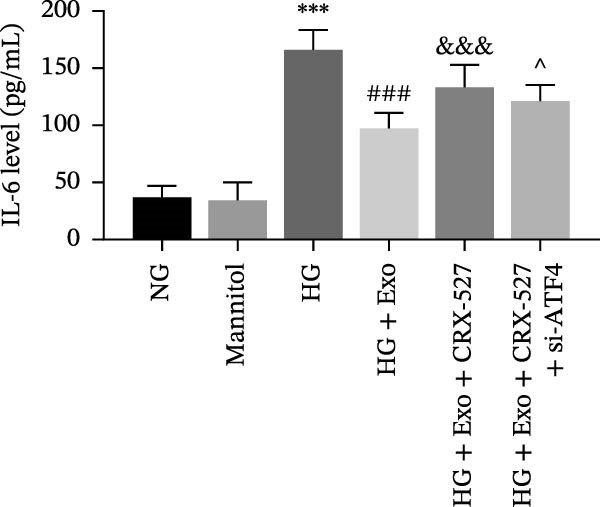
(G)

### 3.4. mBMSCs‐Exo Target and Suppress TLR4 Expression by Delivering Let‐7b‐5p

miRNAs have been recognized as key regulators of DR progression [37]. To identify which miRNAs derived from mBMSCs‐Exo contribute to the suppression of DR, we analyzed the differentially expressed miRNAs in the DR dataset GSE140959 and observed significant downregulation of hsa‐let‐7b expression (Figure 4A,B). Since let‐7b‐5p has been reported to be secreted by stem cells [21] and on the basis of the results in the previous section of this study showing that mBMSCs‐Exo inhibit microglial activation through the TLR4/ATF4 axis, we further investigated whether mBMSCs‐Exo regulate TLR4 expression in microglia through the delivery of let‐7b‐5p. The results revealed that exposure to HG significantly decreased let‐7b‐5p levels in BV‐2 cells, whereas mBMSCs‐Exo intervention restored let‐7b‐5p expression (Figure 4C). Using the ENCORI database, a binding site was predicted between let‐7b‐5p and the 3′‐UTR of TLR4 mRNA (Figure 4D). A dual‐luciferase reporter assay confirmed that cotransfection of agomir‐let‐7b‐5p with the TLR4 WT reporter significantly decreased luciferase activity, whereas transfection with the TLR4 MUT reporter did not affect luciferase activity, indicating that let‐7b‐5p can directly target TLR4 (Figure 4E). To verify the effect of let‐7b‐5p on TLR4, we overexpressed let‐7b‐5p (agomir‐let‐7b‐5p) in BV‐2 cells. RT‐qPCR results demonstrated a marked increase in its expression (Figure S1B). RIP‐qPCR analysis revealed enhanced enrichment of let‐7b‐5p in the promoter region of TLR4 (Figure 4F). Moreover, Western blotting revealed that agomir‐let‐7b‐5p treatment suppressed TLR4 protein expression in the cells (Figure 4G). Together, these findings demonstrate that mBMSCs‐Exo deliver let‐7b‐5p to microglia, where it targets and downregulates TLR4 expression, thereby modulating downstream inflammatory responses.

Figure 4mBMSCs‐Exo target and suppress TLR4 expression by delivering let‐7b‐5p. (A) Heatmap of differentially expressed miRNAs. (B) Volcano plot of differentially expressed miRNAs. (C) Expression of let‐7b‐5p in BV‐2 cells detected by RT‐qPCR. (D) Predicted binding site between let‐7b‐5p and TLR4. (E) Validation of let‐7b‐5p binding to TLR4 via dual‐luciferase reporter assay. (F) Enrichment of let‐7b‐5p at the TLR4 promoter region measured by RIP‐qPCR. (G) TLR4 expression in BV‐2 cells detected by Western blot. Compared with the NG group or the agomir‐NC group,  ^∗^ *p* < 0.05,  ^∗∗^ *p* < 0.01, and  ^∗∗∗^ *p* < 0.001; compared with the HG group, ^##^ *p* < 0.01.

**Figure:**
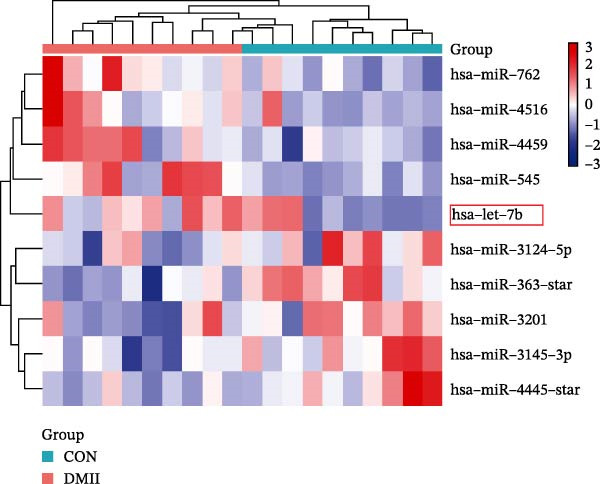
(A)

**Figure:**
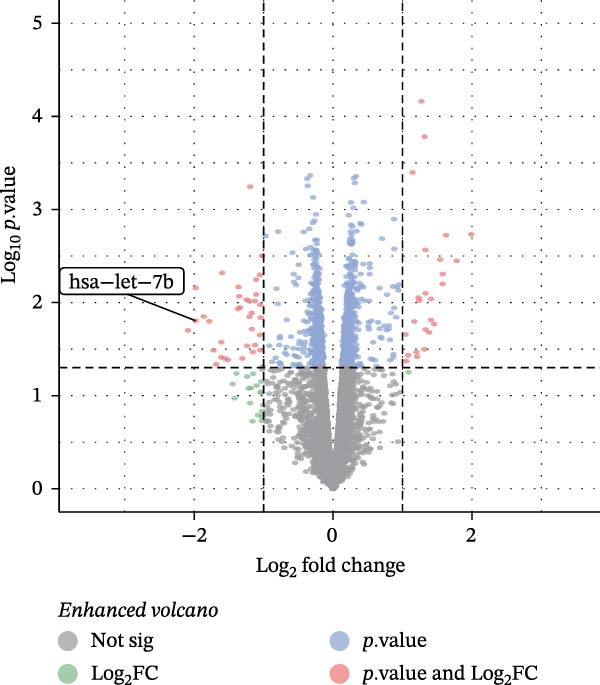
(B)

**Figure:**
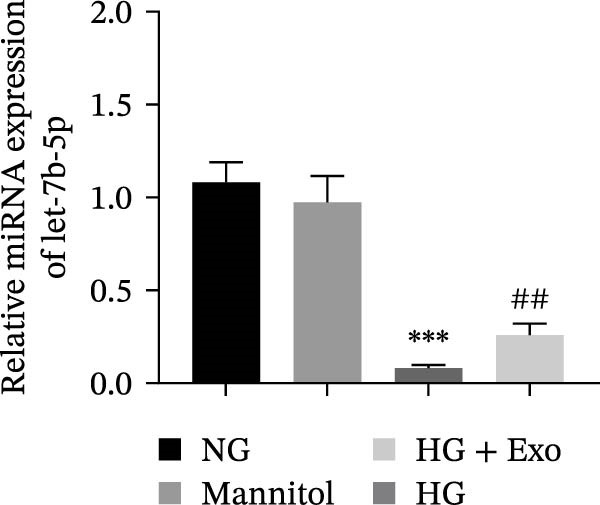
(C)

**Figure:**
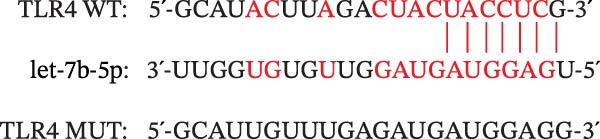
(D)

**Figure:**
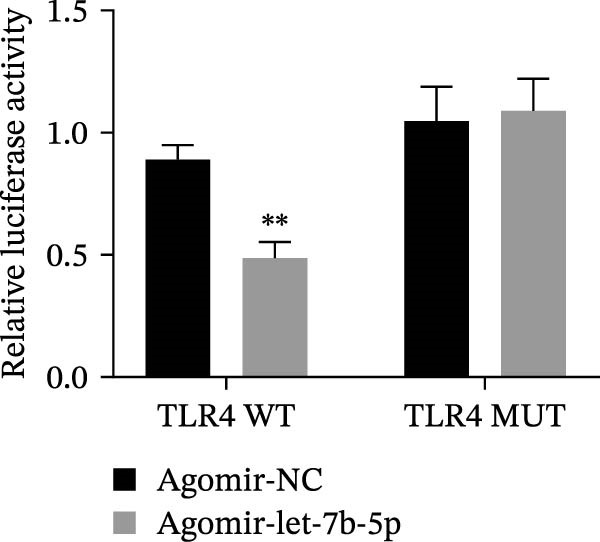
(E)

**Figure:**
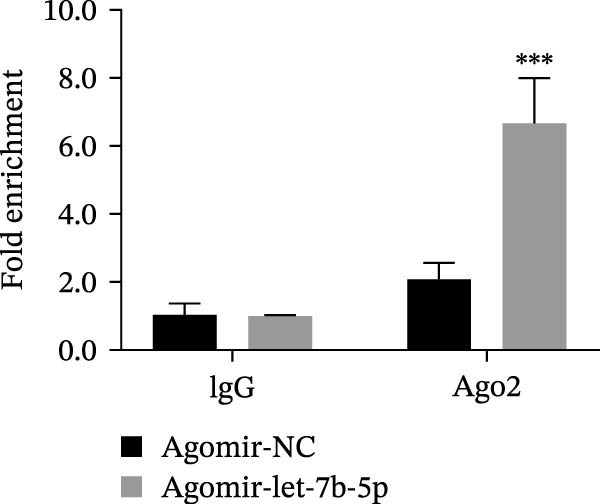
(F)

**Figure:**
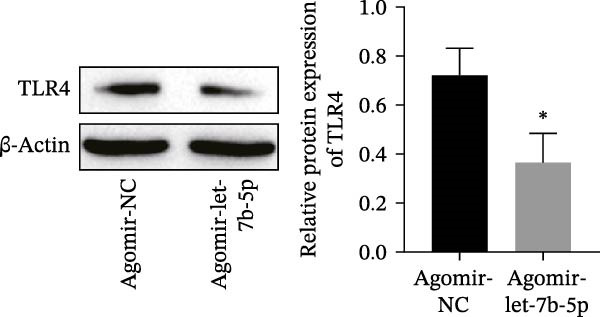
(G)

### 3.5. mBMSCs‐Exo Attenuate HG‐Induced Microglial Activation and Inflammation by Delivering Let‐7b‐5p to Inhibit the TLR4/ATF4 Signaling Axis

To further clarify whether let‐7b‐5p in mBMSCs‐Exo affects microglial activation and inflammation by regulating the TLR4/ATF4 signaling axis, we first overexpressed or knocked down let‐7b‐5p in mBMSCs. The results confirmed that transfection with agomir‐let‐7b‐5p or antagomir‐let‐7b‐5p significantly upregulated or downregulated its expression, respectively (Figure S1C). Exo derived from the corresponding treated mBMSCs (agomir‐let‐7b‐5p‐Exo and antagomir‐let‐7b‐5p‐Exo) were then collected and cultured with BV‐2 cells. Compared with the HG + Exo group, agomir‐let‐7b‐5p‐Exo further increased let‐7b‐5p levels in BV‐2 cells, whereas antagomir‐let‐7b‐5p‐Exo reduced let‐7b‐5p expression (Figure 5A). At the protein level, agomir‐let‐7b‐5p‐Exo enhanced the inhibitory effect of mBMSCs‐Exo on TLR4 and ATF4 expression, whereas antagomir‐let‐7b‐5p‐Exo attenuated this effect (Figure 5B). Immunofluorescence and Western blot analyses further demonstrated that agomir‐let‐7b‐5p‐Exo significantly downregulated the expression of Iba1 and TSPO, whereas antagomir‐let‐7b‐5p‐Exo partially reversed the inhibitory effect of mBMSCs‐Exo (Figure 5C–E). In terms of inflammatory cytokines, agomir‐let‐7b‐5p‐Exo synergistically enhanced the suppressive effect of mBMSCs‐Exo on TNF‐α, IL‐1β, and IL‐6, whereas antagomir‐let‐7b‐5p‐Exo diminished this effect (Figure 5F–H). No statistically significant differences were detected between the mannitol osmotic control group and the NG group in any of the experiments. In summary, mBMSCs‐Exo deliver let‐7b‐5p to microglia, where it targets and inhibits the TLR4/ATF4 signaling axis, thereby alleviating HG‐induced microglial activation and inflammation.

Figure 5BMSC‐Exo suppress HG‐induced microglia through the inhibition of TLR4/ATF4 via let‐7b‐5p. (A) Levels of let‐7b‐5p in BV‐2 cells detected by RT‐qPCR. (B) Expression of TLR4 and ATF4 in BV‐2 cells detected by Western blot. (C) Expression levels of Iba1 and TSPO in BV‐2 cells detected by Western blot. (D–E) Expression levels of Iba1 and TSPO in BV‐2 cells detected by immunofluorescence, scale bar: 10 μm. (F–H) Levels of the inflammatory cytokines TNF‐α, IL‐1β, and IL‐6 in the supernatant of BV‐2 cells measured by ELISA. Compared with the NG group,  ^∗∗∗^ *p* < 0.001; compared with the HG group, ^###^ *p*  < 0.001; compared with the HG +Exo group, ^&^ *p* < 0.05, ^&&^ *p* < 0.01, and ^&&&^ *p*  < 0.001.

**Figure:**
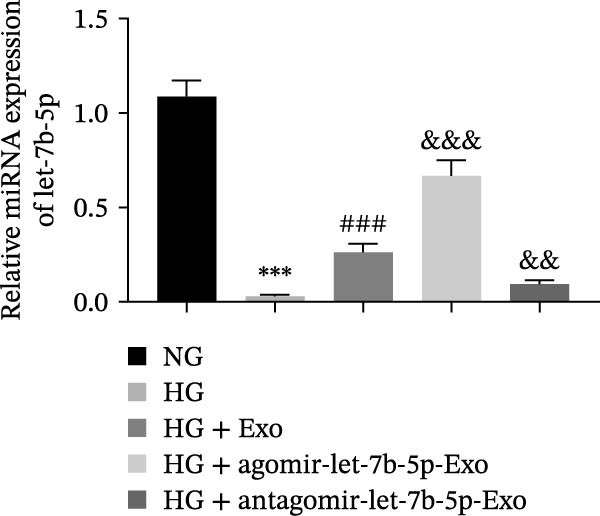
(A)

**Figure:**
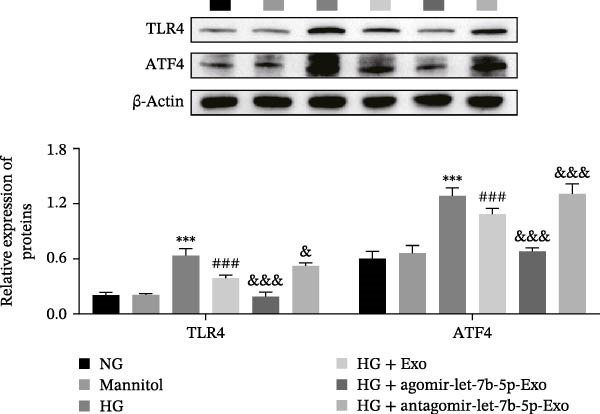
(B)

**Figure:**
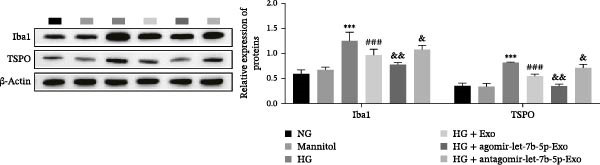
(C)

**Figure:**
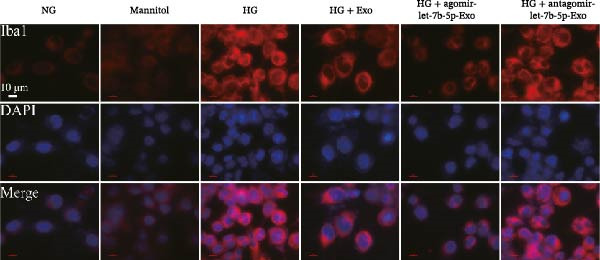
(D)

**Figure:**
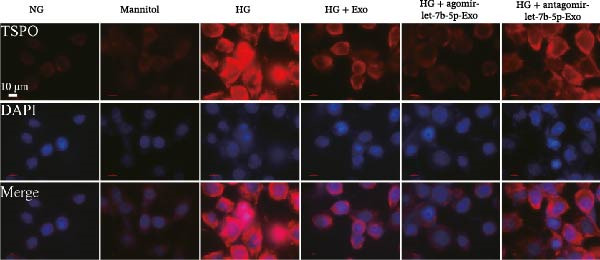
(E)

**Figure:**
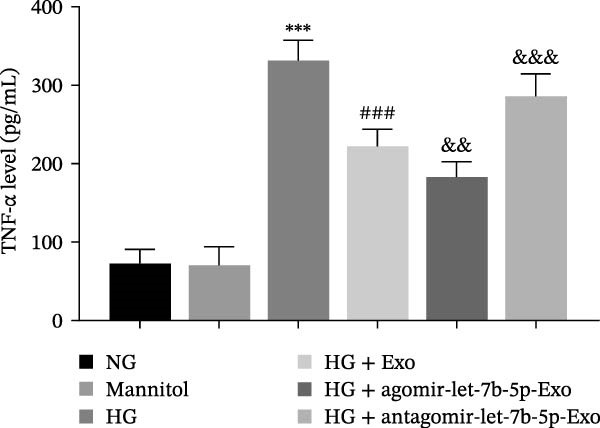
(F)

**Figure:**
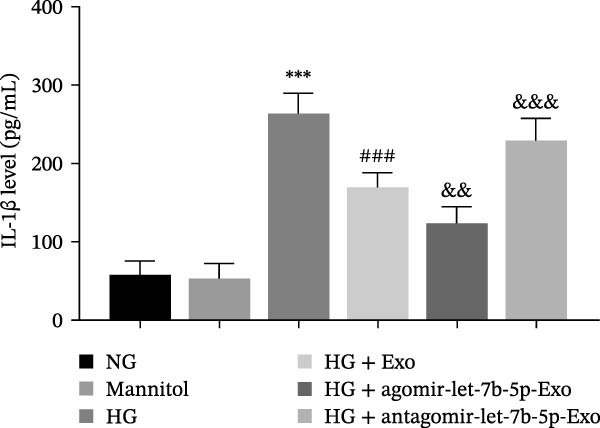
(G)

**Figure:**
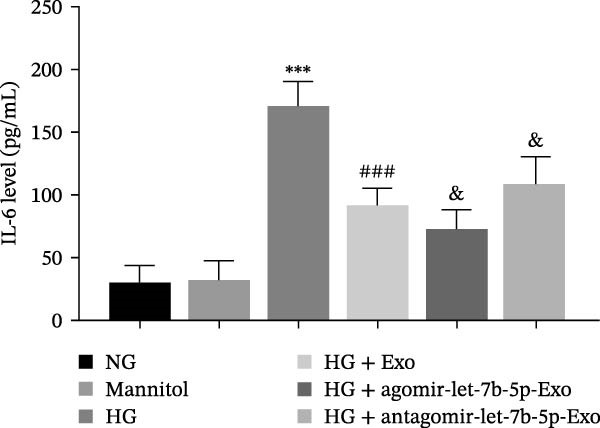
(H)

### 3.6. mBMSCs‐Exo Attenuate Retinal Injury and Inflammation in DR Mice Through Delivery of Let‐7b‐5p

To validate the role of let‐7b‐5p in mBMSCs‐Exo in DR in vivo, we examined molecular expression and pathological changes in mouse retinal tissues. Consistent with the in vitro findings, let‐7b‐5p expression was downregulated, while TLR4 and ATF4 expressions were upregulated in the retinas of DR mice. Treatment with mBMSCs‐Exo reversed this trend, significantly increasing let‐7b‐5p levels and suppressing TLR4 and ATF4 expression. Notably, compared with conventional mBMSCs‐Exo, agomir‐let‐7b‐5p‐Exo had a stronger effect, whereas antagomir‐let‐7b‐5p‐Exo partially attenuated the protective effect of mBMSCs‐Exo (Figure 6A,B). HE staining revealed that DR mice exhibited a thinning of the retinal layer, a disorganized structure, and a sparse cell arrangement. Following mBMSCs‐Exo treatment, the retinal thickness increased, the structure became more compact, and the cells were arranged more orderly. This improvement was more pronounced in the agomir‐let‐7b‐5p‐Exo group, whereas these beneficial changes were partially reversed in the antagomir‐let‐7b‐5p‐Exo group (Figure 6C). TUNEL staining further demonstrated that mBMSCs‐Exo significantly reduced retinal apoptosis in DR mice. This antiapoptotic effect was enhanced in the agomir‐let‐7b‐5p‐Exo group but weakened in the antagomir‐let‐7b‐5p‐Exo group (Figure 6D). Western blot and immunofluorescence staining revealed elevated expression of Iba1 and TSPO in the retinas of DR mice, which was effectively suppressed by mBMSCs‐Exo treatment. The inhibitory effect was stronger with agomir‐let‐7b‐5p‐Exo, whereas antagomir‐let‐7b‐5p‐Exo partially counteracted the effect of mBMSCs‐Exo (Figure 6E–G). Moreover, mBMSCs‐Exo treatment significantly decreased the serum levels of the inflammatory cytokines TNF‐α, IL‐1β, and IL‐6 in DR mice. This reduction was further enhanced in the agomir‐let‐7b‐5p‐Exo group but diminished in the antagomir‐let‐7b‐5p‐Exo group (Figure 6H–J). These results indicate that mBMSCs‐Exo deliver let‐7b‐5p to target and inhibit the TLR4/ATF4 signaling pathway, thereby suppressing microglial activation, alleviating inflammation, and ultimately ameliorating DR pathology.

Figure 6mBMSCs‐Exo attenuate retinal injury and inflammation in DR mice through delivery of let‐7b‐5p. (A) Expression of let‐7b‐5p in mouse retinal tissue detected by RT‐qPCR. (B) Expression of TLR4 and ATF4 in mouse retinal tissue detected by Western blot. (C) Retinal tissue injury assessed by HE staining, scale bar: 25 μm. (D) Apoptosis level in mouse retinal cells detected by TUNEL staining. (E) Expression of the microglial activation markers Iba1 and TSPO in mouse retinal tissue detected by Western blot. (F–G) Expression of the microglial activation markers Iba1 and TSPO in mouse retinal tissue detected by immunofluorescence. (H–J) Levels of the inflammatory cytokines TNF‐α, IL‐1β, and IL‐6 in the serum of DR mice measured by ELISA. Compared with the control group,  ^∗∗∗^ *p* < 0.001; compared with the DR group, ^###^ *p* < 0.001; compared with the DR + Exo group, ^&^ *p* <  0.05, ^&&^ *p*  < 0.01, and ^&&&^ *p*  < 0.001.

**Figure:**
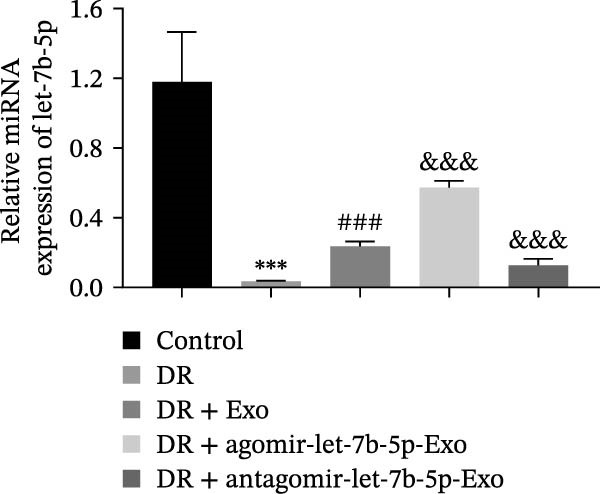
(A)

**Figure:**
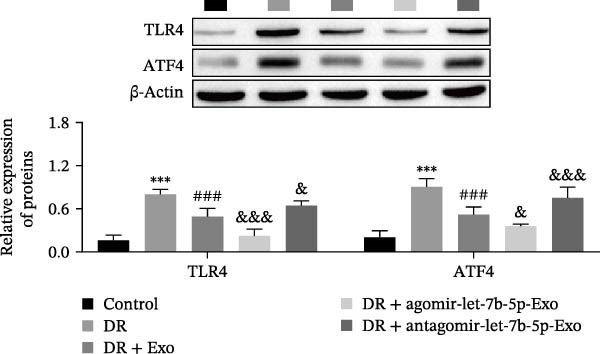
(B)

**Figure:**
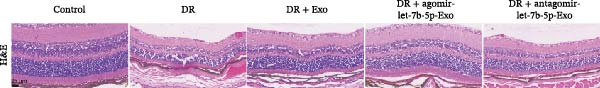
(C)

**Figure:**
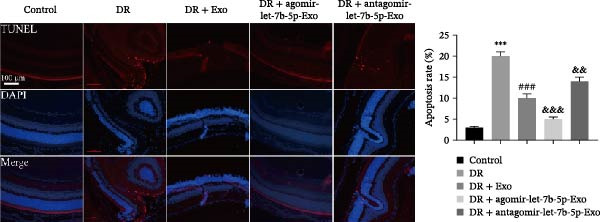
(D)

**Figure:**
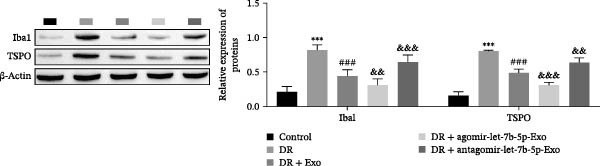
(E)

**Figure:**
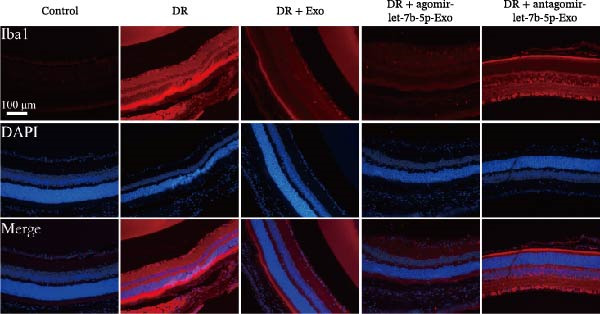
(F)

**Figure:**
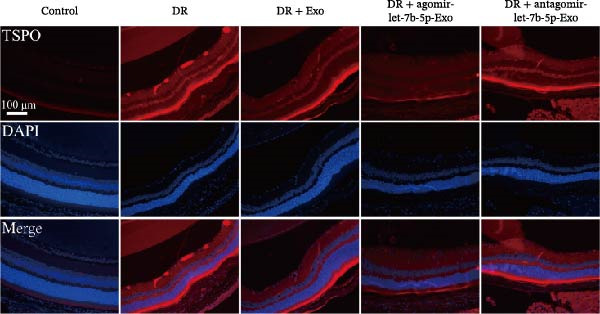
(G)

**Figure:**
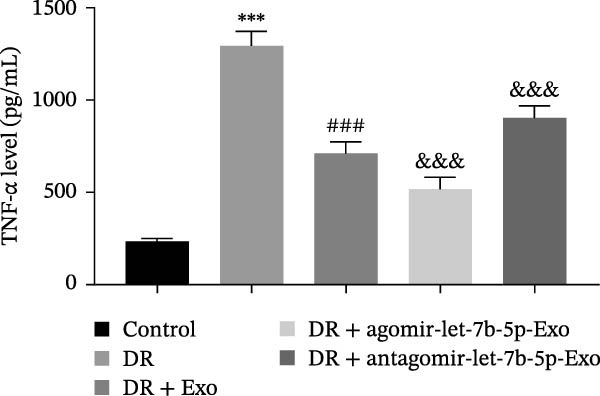
(H)

**Figure:**
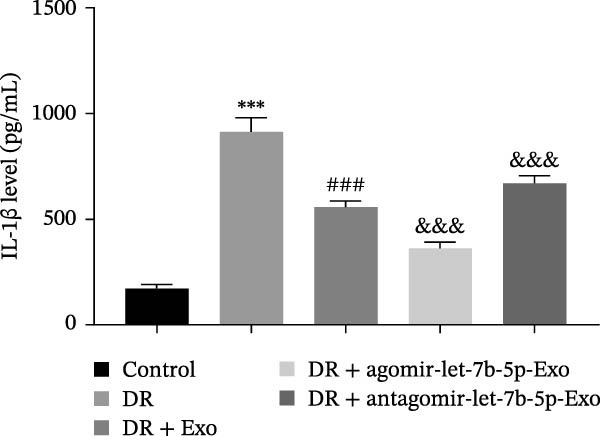
(I)

**Figure:**
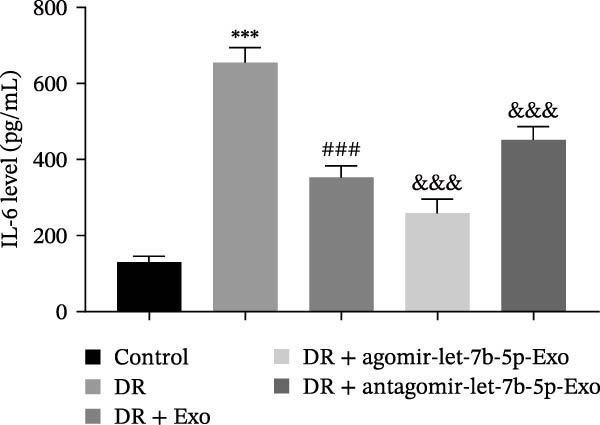
(J)

## 4. Discussion and Conclusions

DR, a severe complication of DM, significantly impairs patients’ vision and has become a leading cause of blindness in both elderly individuals and the global working‐age population [3]. This study revealed that let‐7b‐5p delivered by mBMSCs‐Exo can alleviate DR by suppressing the activation of the TLR4/ATF4 signaling axis, thereby inhibiting microglial activation and the associated inflammatory response. Our findings highlight the strong potential of mBMSCs‐Exo–mediated let‐7b‐5p delivery in mitigating the progression of DR.

Microglia are immune cells that reside in the retinal plexiform layer and play important roles in maintaining retinal homeostasis [6]. In DR, persistent hyperglycemic infiltration induces increased expression of inflammatory chemical mediators such as cytokines, chemokines, and growth factors in retinal tissue, leading to a proinflammatory environment in the retina before the clinical symptoms of DR appear [38]. This inflammatory environment can activate microglia. Importantly, these phenotypic changes in microglia are considered to promote the development of DR [7]. Studies by Chen et al. [39] have shown that activated microglia release large amounts of cytokines such as IL‐1β and amplify angiogenesis, ultimately contributing to the occurrence of DR. In the research by Tang et al. [40], overactivated microglia caused damage to the blood–retinal barrier, which is also a key factor in the progression of DR. Consistent with previous findings, we observed elevated microglial activation levels in HG‐treated BV‐2 cells and retinal tissues of DR mice, along with upregulation of TNF‐α, IL‐1β, and IL‐6, indicating that microglial activation and increased inflammation are associated with the progression of DR.

In recent years, the therapeutic potential of MSCs‐Exo in DR has garnered increasing attention. Multiple studies have confirmed that MSCs‐Exo can ameliorate the pathological progression of DR through various mechanisms, including by inhibiting endothelial–mesenchymal transition and abnormal angiogenesis [41] and delaying the senescence of retinal pigment epithelial cells to maintain retinal structural and functional integrity [42]. However, whether BMSCs‐Exo influence DR progression by regulating microglial activation remains insufficiently explored. This study revealed that mBMSCs‐Exo can be efficiently taken up by BV‐2 microglia and significantly inhibit HG‐induced microglial activation; reduce the levels of proinflammatory factors such as TNF‐α, IL‐1β, and IL‐6; and subsequently alleviate retinal damage in DR mice. These findings reveal a mechanism through which mBMSCs‐Exo improve DR through the modulation of inflammation. Notably, recent studies on the role of EVs in regulating tissue fibrosis and inflammatory responses have shown that mesenteric adipose tissue‐derived Exo (MAT‐Exo) deliver the TINAGL1 protein to activate the TGF‐β/SMAD4 signaling pathway, thereby exacerbating intestinal fibrosis in the chronic inflammatory disease Crohn’s disease (CD) [43]. These findings are in striking contrast to our findings, collectively highlighting the bidirectional regulatory roles of Exo in disease pathogenesis. These studies provide crucial theoretical foundations for developing targeted therapeutic strategies based on Exo.

Research has indicated that TLR4 expressed by microglia acts as a key pattern recognition receptor capable of recognizing both pathogens and endogenous danger signals, thereby initiating adaptive immune responses [44]. Upon activation, TLR4 significantly promotes the production of proinflammatory mediators through downstream signaling [45]. Notably, Xue et al. [46] reported that BMSCs‐Exo can inhibit TLR4 expression, thereby blocking microglial activation and inflammatory responses. These findings align with those of the study by Zhang et al. [31], which confirmed that TLR4 activation induces nuclear translocation of ATF4, further promoting the secretion of inflammatory factors such as IL‐6 and IL‐8 by monocytes. The TLR4/ATF4 signaling axis has been shown to be involved in the progression of various diseases, such as the development of metabolic dysfunction‐associated fatty liver disease in a maternal obstructive sleep apnea rat model [47] or the exacerbation of inflammatory responses in *Aspergillus fumigatus* keratitis [48]. However, whether BMSCs‐Exo exert anti‐DR effects by regulating this signaling axis remains unclear. The results of this study revealed that the expression of TLR4 and ATF4 was significantly upregulated in BV‐2 cells under HG conditions and in the retinas of DR mice, whereas mBMSCs‐Exo intervention reversed these effects. Key experiments confirmed that treatment with a TLR4 activator partially counteracted the protective effects of mBMSCs‐Exo, reactivated microglia, and increased the release of inflammatory factors such as TNF‐α, IL‐1β, and IL‐6, whereas knockdown of ATF4 attenuated the effects of CRX‐527. These results reveal that mBMSCs‐Exo protect against DR by inhibiting the TLR4/ATF4 signaling axis, thereby suppressing microglial activation and inflammatory responses.

Research has demonstrated that the therapeutic effects of BMSCs‐Exo on DR are mediated primarily through their miRNA cargo [16, 17]. Notably, let‐7b‐5p, a member of the let‐7 miRNA family, has been shown to regulate microglial activation under neurodegenerative stimuli, with its downregulation potentially promoting myeloid cell activation and accelerating neurodegenerative processes [49, 50]. These findings are consistent with those of Mandolesi et al.’s [23] study on multiple sclerosis (a chronic inflammatory neurodegenerative disorder), which revealed a significant correlation between let‐7b‐5p deficiency and disease progression. Importantly, Liu et al. [25] reported that Exo derived from iPSC‐NSCs can suppress microglia/macrophage activation and subsequent inflammatory responses via let‐7b‐5p delivery. Notably, existing studies have shown that let‐7b‐5p can alleviate macrophage‐mediated colitis by targeting TLR4 [32]. In this study, bioinformatics analysis revealed decreased let‐7b‐5p expression in DR along with potential targeting relationships with TLR4, which were subsequently validated through dual‐luciferase reporter assays and RIP experiments. Mechanistic investigations demonstrated that let‐7b‐5p overexpression could enhance the therapeutic efficacy of mBMSCs‐Exo, while its knockdown partially attenuated these protective effects. These findings collectively reveal that mBMSCs‐Exo exert their DR therapeutic effects by delivering let‐7b‐5p to specifically inhibit the TLR4/ATF4 signaling axis, thereby modulating microglial activation and neuroinflammatory responses.

In summary, our study demonstrated that mBMSCs‐Exo can effectively deliver let‐7b‐5p and alleviate microglial activation and related inflammatory responses by inhibiting the TLR4/ATF4 signaling pathway, thereby providing a novel therapeutic approach to blocking DR progression. It should be noted that future research could be expanded in the following aspects: First, incorporating functional visual assessments such as electroretinography (ERG) would help to more comprehensively reveal the direct effects of this strategy on visual function improvement. Second, systematic analysis of exosomal cargo (e.g., RNA sequencing or proteomics) may further elucidate potential synergistic interactions between let‐7b‐5p and other components. Furthermore, validation in human primary cells or clinically relevant samples would provide stronger support for the clinical translation of this mechanism. These follow‐up studies will facilitate the advancement of exosome‐based therapeutic strategies toward clinical application.

## Author Contributions

All the authors contributed substantially to this manuscript. Conceptualization: Yepin Zhang and Yiyi Luo. Data curation: Peiqi Chen, Libo Zhang, and Jian Han. Formal analysis: Ling Wang, Jian Han, and Hong Xu. Investigation: Heng Luo. Methodology: Ling Wang, Jian Han, and Hong Xu. Project administration: Yepin Zhang and Yiyi Luo. Resources: Heng Luo. Software: Peiqi Chen and Libo Zhang. Supervision: Heng Luo. Validation: Yepin Zhang. Visualization: Yiyi Luo. Writing – original draft: Yepin Zhang and Yiyi Luo. writing – review and editing: Heng Luo.

## Funding

The authors have nothing to report.

## Disclosure

All the authors have read and agreed to the published version of the manuscript.

## Ethics Statement

The animal experimental procedures were approved by the Medical Ethics Committee of Chuxiong Yi Autonomous Prefecture People’s Hospital (No. 2022‐11) and were performed in accordance with the ARRIVE guidelines for the care and use of laboratory animals.

## Conflicts of Interest

The authors declare no conflicts of interest.

## Supporting Information

Additional supporting information can be found online in the Supporting Information section.

## Supporting information


**Supporting Information** This study includes one supporting figure: Figure S1. Confirmation experiments for transfection efficiency.

## Data Availability

Data are available upon request from the authors.
