# Supplementary material for: Bone Marrow Mesenchymal Stem Cell‐Derived Exosomal Let‐7b‐5p Reduces High Glucose‐Induced Microglial Activation and Inflammation Through TLR4/ATF4
Source: Mediators Inflamm. 2026 Feb 10;2026:7251718. doi: 10.1155/mi/7251718 (PMC12887830; doi:10.1155/mi/7251718)
Supplement: Supplementary file 1 — Supporting Information This study includes one supporting figure: Figure S1. Confirmation experiments for transfection efficiency. [file MI-2026-7251718-s001.docx]

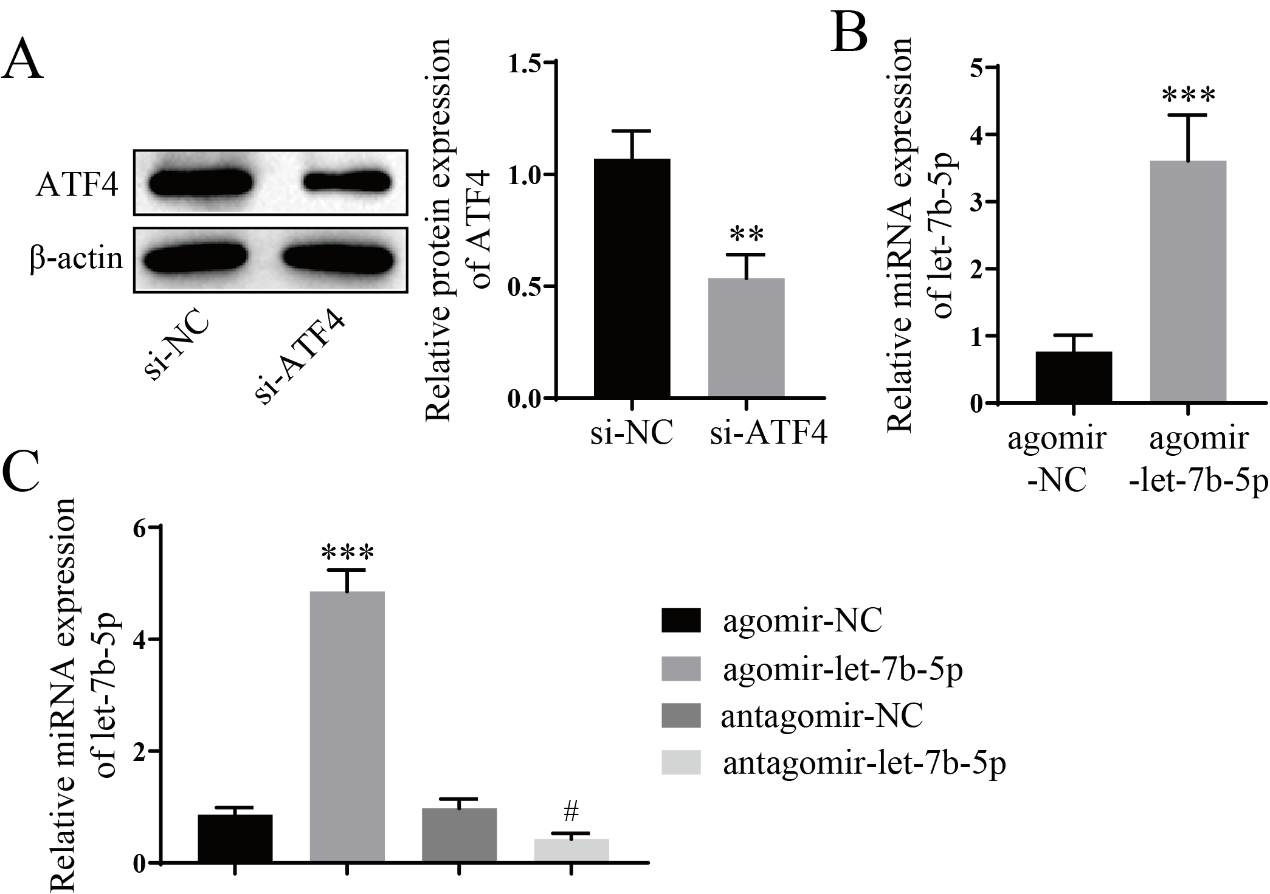


**Supplementary Figure 1 Confirmation experiment for transfection efficiency**

A: Western blot detection of ATF4 expression to confirm the transfection efficiency of si-ATF4; B: RT-qPCR detection of let-7b-5p expression in BV-2 cells to confirm transfection efficiency; C: RT-qPCR detection of let-7b-5p expression in mBMSCs to confirm transfection efficiency.
